# Ostracized but why? Effects of attributions and empathy on connecting with the socially excluded

**DOI:** 10.1371/journal.pone.0201183

**Published:** 2018-08-03

**Authors:** Michael J. Bernstein, Zhansheng Chen, Kai-Tak Poon, Jacob A. Benfield, Henry K. S. Ng

**Affiliations:** 1 Department of Psychology, Penn State University Abington, Abington, Pennsylvania, United States of America; 2 Department of Psychology, The University of Hong Kong, Hong Kong Island, Hong Kong; 3 Department of Psychology, The Education University of Hong Kong, New Territory, Hong Kong; Universite de Bretagne Occidentale, FRANCE

## Abstract

The present research examined people’s responses towards others’ exclusion experience. The authors predicted that both causal attributions and empathy would mediate whether people affiliate with a victim of an ambiguous exclusion experience. Perceivers observing another’s exclusion (relative to inclusion) without clearly announced reasons chose to affiliate with the target and this was mediated by increased external attributions for the exclusion (Studies 1a, 1b, 2). When the attributions people made for the exclusion of a target was experimentally manipulated, internal attributions decreased desire for affiliation relative to external or ambiguous attributions, and this was mediated by differences in empathy for the target (Study 3). Further, external attributions arisen from perceiving a causally unclear exclusion leads to an empathetic response which results in an increased desire to affiliate with the target (Study 4). Future directions on perceptions of those who have been excluded are discussed.

## Introduction

Myriad studies have been conducted examining the consequences of social exclusion [1,2]. Research has shown that individuals who lack belonging (e.g., due to rejection) often respond in very prosocial, seemingly adaptive ways including increases in social compensatory behavior [3], the desire to affiliate [4], and the ability to accurate and quickly identify facial expressions of emotions [5]. Similarly, other research has found that individuals can become aggressive after social exclusion [6,7], exhibit deficits in cognitive [8] and self-regulatory abilities [9], and behave dishonestly [10]. Generally, social exclusion is a painful experience (see [11]), having effects on both physical pain [12,13] as well as emotional responses [14].

While considerable attention has been paid to such outcomes of social exclusion, little work has been done on how people perceive and respond to others’ experience of social exclusion. This is perhaps surprising, given the ubiquitous and detrimental nature of social exclusion experiences and the situations under which it often occurs; social exclusion can be used to punish group members who threaten group goals either by choice or by lack of ability (e.g., cheaters, non-reciprocators; see [15]. Thus, it stands to reason that perceivers have a motivation to think about *why* an individual was the target of social exclusion; answers to this question could play a critical role in determining whether a person should continue to experience exclusion or should be offered an opportunity to (re)affiliate and whether a perceiver should invest resources in the victim of rejection.

### Attribution of others’ experience of exclusion

Social exclusion is often ambiguous or uncertain [16,17]. As noted by Williams [18], “ostracism is inherently ambiguous” (p.142). The victims of ostracism may not be certain whether they are actually ostracized or whether their experience is due to misunderstanding or communication difficulties; even when people do face the reality of ostracism, they may still uncertain about the underlying motivations. Observers may face the same causal uncertainty when witnessing an episode of ostracism.

Researchers have long asserted that human beings are intuitive scientists, routinely developing explanations, or naïve theories for both their own behaviors and the behaviors of others. Individuals work to infer the cause of why individuals behave as they do in various situations (e.g., [19–21]). Heider’s early work [21,22] identifies a primary distinction for explaining the causes of behavior; generally, attributions for behaviors are identified as being either internal or external. Internal attributions (i.e., dispositional attributions) are those in which an aspect of the self is perceived as the causal agent of an event (e.g., the reason why a person volunteers for charity is being they are a caring person). Conversely, external attributions (i.e., situational attributions) are those in which something outside the person, such as the environment or another person, is perceived as the cause of behavior (e.g., the reason why a person volunteers for charity is because they are trying to build a stronger college resume). Individuals make internal attributions when *aspects of the self* are perceived as covarying along with a particular outcome, yet make external attributions when an *external factor* is perceived as covarying with an event or outcome [21].

Human beings have considerable interest in forming bonds with others [23], but if another has been socially excluded, peoples’ motivation to affiliate must be tempered with a desire to know why an individual was excluded from their group. Forming a causal attribution should be paramount in deciding whether a perceiver should approach or avoid the excluded person. If individuals make internal attributions for another’s exclusion, they should have less desire to affiliate with the excluded target; again, exclusion has long been a tactic to punish individuals who are poor group members, and thus if perceivers assume that an excluded individual is inherently and dispositionally responsible for their exclusion, they should be less willing to affiliate with the rejected target. In these situations, a target behaved in a manner that led to exclusion and furthermore, the cause of this behavior was deemed a dispositional characteristic of the rejected; thus, it stands to reason that this person deserved their ‘punishment’ and would be likely to behave in a similar negative fashion in the future. If, however, perceivers make external attributions for the exclusion of another, they should be more willing to affiliate with the target, precisely because the exclusion is not necessarily indicative of anything about the victim that would be a cue to their being a poor affiliation partner. Specifically, the exclusionary event was caused primarily by factors outside of the characteristics of the rejected, indicating that this individual may not be inherently flawed, or unworthy of affiliative attention.

### The mediational role of empathy

While we argue that the type of attribution is an antecedent factor that influences an individuals’ decision to avoid or affiliate with a previously rejected individual, we hypothesize that the psychological mechanism that determines this response is differential empathy towards the rejected target, which is itself determined by the attributions formed.

Empathy is defined as a response to another person’s condition (e.g., physical, emotional) that is other-oriented [24,25]. Feelings of sympathy and compassion for another person’s plight can result in their attempting to aid the person, perhaps by reducing their distress of need. Hoffman [26] described that empathy results in people having a concern for others’ well-being and thus can lead to their actively engaging in behaviors to aid the person in need. Numerous studies have shown that individuals who feel empathy for another are more likely to engage in a host of positive behaviors towards that person including helping, more positive attitudes, and greater self-disclosure (e.g., [27–30]).

There are good reasons to think that exclusionary experiences that are due to factors of the situation (rather than the disposition of the victim) may elicit empathy from perceivers which may in turn result in their affiliating with them. Research has demonstrated that social relationships for which there is mutual respect and equal opportunities exhibit a norm of equality (e.g., [31]). When this norm of equality is violated for no justifiable reason, the victims of such treatment are viewed sympathetically [32], and of particular interest for the current work, perceivers of an episode of undeserved exclusion both recognized the distress that another felt due to the ostracism and felt as though they were experiencing exclusion themselves [33]. As stated previously, because empathy is related to helping, we believe that individuals who perceive another as excluded due to external reasons will empathize with the target and will help them by offering them affiliation. These predictions are consistent with research focusing how people respond to their group members’ experience of ostracism [34]. In particular, Wesselmann and colleagues [34] found that people are likely to help an ostracized group member, unless they perceive the ostracized member as a burden to the group’s performance.

Our hypotheses are further supported by additional research on the relationship between empathy and attributions. According to attribution theory [35], when the cause of a person’s need is beyond their control (e.g., external to their person), people experience an affective response (e.g., pity, sympathy, empathy) for the person and this mediates their increased helping. In research supporting this logic, Meyer and Mulherin [36] had participants read a hypothetical scenario in which they were approached by an acquaintance for financial help paying his/her rent. Various aspects of the scenarios were manipulated by describing the person’s employment history. Most importantly for the current investigation, they manipulated the perceived cause of the person’s need as being either due to relatively internal (e.g., chose to miss work when not in the mood) or external (e.g., was unable to work due to high unemployment rate) reasons. Among other dependent variables, participants rated their emotional reaction to the target as well as their willingness to help. The results indicated that when the person’s plight was due to more external factors, participants felt greater empathy for the target. Further, participants were also more willing to help those for whom relatively external factors were the cause of their need. Finally, a path analysis revealed that the effect that external factors had on people’s willingness to help the person in need was mediated by their affective (e.g., empathetic) response. Other work supports the claim that when a person’s plight is attributable to external factors, this generates an empathetic response that in turns leads to helping (e.g., [37–39]).

Thus, we believe that external attributions themselves result in increased empathy that in turn results in a greater desire to affiliate (a prosocial behavior; see [4]) with the rejected person. This empathetic response may not be present for a victim whose exclusion is internally attributed and thus may elicit a “they deserved it” attitude. Thus, we further hypothesize that the extent to which individuals empathize with the victim will mediate the relationship between their making external attributions and their desire to affiliate with the target.

We think these hypotheses are well grounded in the extant literature. An astute reader would be correct to think that it has been established that external attributions for misfortunes lead to greater empathy for the victims of such consequences. We believe, however, it remains unclear how people make internal or external attributions following witnessing social exclusion and whether the chain of events previously shown (whereby external attributions lead to greater empathy) occurs for those whom have been excluded, an act which itself can be used by groups as a signal to others that a member is undesirable (e.g., [40]). Thus, we believe examining these hypotheses will shed important light on a question as of yet unanswered.

We examined these hypotheses in the following five studies. In all studies, the right of participants were protected. All studies were subject to the Penn State University Office of Research Protections who approved the studies. Informed consent was given online at the start of all studies and participants had to either select they agreed to participate or elected not to do so following reading the consent. This processed was approved by the Office of Research Protections.

## Study 1a

Study 1a aimed to provide an initial test to our hypothesis that observing another’s exclusion without clearly announced reasons would lead perceiver to attribute the exclusion to external factors (rather than factors internal to the target of exclusion) which further promotes perceivers’ intention of affiliating with the target.

Participants read a supposed chat log between three supposed students participating in an online discussion for a class research requirement. The three targets were Sam, Jacob, and Robert. While the chat logs all began similarly, in the included chat log, Robert (the target in this study) is continually involved in the conversation with individuals responding to his questions and asking questions of him. In the excluded condition, however, Robert is eventually ignored entirely, with no responses to his questions and no inclusion of him in the conversation. Importantly, we intentionally left the reason for this ambiguous; Robert neither says anything which indicates he has done something to bring on the exclusion nor is there some obvious external factor resulting in the exclusion. We then examined perceptions of exclusion, the extent to which people made internal or external attributions for the exclusion, and the extent to which participants would like to affiliate with Robert.

### Method

#### Participants and design

G*power analyses [41] for an independent sample t-test showed that a sample size of 128 participants were needed to detect a medium effect (d = .50) with power of .80. One-hundred and nineteen individuals from the United States (54 Female, *M*_*Age*_ = 31.73, *SD*_*Age*_ = 11.52) participated in an online study via Mechanical Turk (e.g., [42]; see [43] for a review) in exchange for .25 USD. Participants were 75.6% White, 5.9% Hispanic, 5.9% Black, 5.9% Asian, and 6.7% Other. All participants were exposed to an Inclusion or Exclusion condition. Sex did not qualify any of the results described below and thus it will not be discussed further.

#### Procedure

All participants were given consent online and then read a chat log involving a supposed interaction occurring between three students (Sam, Jacob, and Robert) at a major Midwestern university. Participants were randomly assigned to either read a chat log in which all three targets were included equally or one where one of the targets (i.e., Robert) was excluded from the conversation.

Afterwards, all participants made ratings of Robert. The questions are described below. The order of the question sets was counterbalanced across participants.

Participants responded to three Likert-type questions assessing how included/excluded Robert was in the conversation. Example items include “Robert was included in the conversation,” and “Robert was ignored during the conversation.” These were assessed on *1-Strongly Disagree* to *9-Strongly Agree* and composite scores were formed for the items (α = .95) such that higher numbers indicated greater inclusion of the target.

We included three items assessing the extent to which participants thought the treatment of Robert was due to internal or external factors (items adapted from [44]). Participants were asked to identify “Does the way Robert was treated reflect an aspect of himself or an aspect of the situation,” “Is the cause of the way Robert was treated in the interaction outside of him or inside of him,” and “Is the cause of the way Robert was treated something about him or something about the others?” These were all responded to on 9-point Likert type scales were each end point was the polar ends of the question (i.e., 1-Something about Others, 9-Something about him). We formed a composite after reverse coding where appropriate (α = .67) such that higher numbers indicated more external attributions for the behavior of the target.

Participants also responded to five additional questions measuring their desire for affiliation with Robert. Participants were asked to assess the extent to which they were interested in learning more and making friends with Robert (1-Not at all interested to 9-Very much interested) as well as how likely they would be to invite him to a party, choose to work with him on a team project, and engage him in a conversation (1-Not at all likely to 9-Extremely likely). We again formed a composite (α = .87) such that higher numbers indicated a greater desire for affiliation with that particular target.

Finally, demographic information was assessed and participants were given an online debriefing and thanked for their time.

### Results and discussion

#### Manipulation check

We conducted an independent sample t-test to examine whether participants were sensitive to the differences in inclusionary status of Robert in the two conditions. This revealed a significant difference, *t*(117) = 13.20, *p* < .001, *d* = 2.40. When Robert was excluded (*M* = 2.81, *SD* = 2.20), he was perceived as being less included than he was in the inclusion condition (*M* = 7.51, *SD* = 1.68). Thus, our manipulation was successful.

#### Internal/External attributions

We next conducted an independent sample t-test on the composite measure of external attributions. This too revealed a significant effect, *t*(117) = 6.38, *p* < .001, *d* = 1.16. When Robert was excluded (*M* = 6.85, *SD* = 1.77), perceivers made more external attributions than when he was included (*M* = 5.06, *SD* = 1.29). Thus, it appears that perceivers give the “benefit of the doubt” as it were and default towards more external attributions for ambiguous exclusions.

#### Desire for affiliation

We next examined whether differences in inclusionary status also led to differences in the desire to affiliate with Robert. Again, an independent sample t-test revealed a significant difference between the two conditions, *t*(117) = 2.22, *p* = .028, *d* = .41. Here, when Robert was excluded (*M* = 6.01, *SD* = 1.91), he was rated as an even more desirable affiliation partner than when he was included (*M* = 5.28, *SD* = 1.68).

#### Mediation analysis

**Fig 1:**
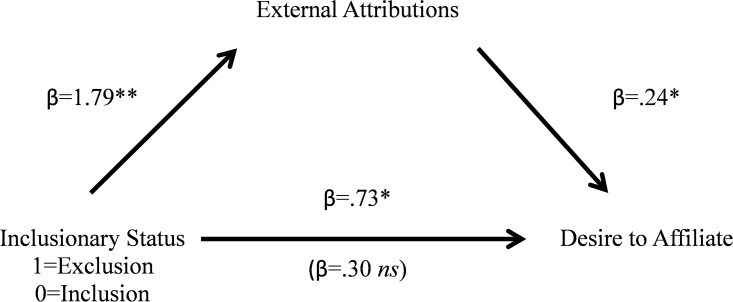
External attribution mediates the linkage between inclusionary status on desire to affiliate (Study 1a). **p< = .001, *p< = .05.

We wanted to examine whether or not the extent to which individuals made more external attributions for Robert’s situation mediated the relationship between the perceived exclusion and the desire for affiliation. A bootstrapping mediation analysis [45] with 5000 iterations was conducted to examine this hypothesis. The experimental condition was coded as 1 (ostracism) or 0 (inclusion). The 95% confidence interval for the indirect path coefficient excluded zero (0.037 to 0.89), suggesting a significant indirect effect (see Fig 1 for betas and *p*-values).

We hypothesized that when exclusion was perceived as being due to external factors, individuals would be more willing to affiliate with the excluded person. We found support for this hypothesis in this first study; when Robert was excluded for ambiguous reasons, individuals perceived his plight to be due more to external and less to internal factors. Further, participants showed more interest in affiliating with Robert when he was excluded. Finally, the extent to which they made external attributions for his exclusion predicted their desire to affiliate with him.

While this supports our contention that attributions play a key role in our perceptions of excluded targets, this study is not without flaws. To begin, we examined responses towards male targets only. While we do not have a strong reason to believe that perceptions of female targets would differ in a similar situation, it is certainly a possibility worth examining; because females more often employ social aggression in intra-sex contexts (as opposed to men who are more likely to use physical aggression, see [46] for review), and because social exclusion is a form of social aggression, it is possible that the desire for affiliation observed when Robert is excluded may be due to a perceived mismatch between Robert’s sex and the aggression used against him. We wished to make certain this effect was not due to an artifact of the sex of the targets. Further, given the new nature of this research, it would be important to replicate the observed effect to strengthen confidence in our findings.

## Study 1b

Study 1b aimed to replicate Study 1a by examining how people respond to a socially excluded female target. As in Study 1, participants read a chat log between 3 students participating in an online discussion. The three targets were Sarah, Jessica, and Rebecca. Rebecca was either included or exclusion during the conversation as in Study 1. We measured the extent to which people made internal or external attributions for the exclusion and the extent to which participants would like to affiliate with Rebecca.

### Method

#### Participants and design

As in Study 1a, a sample size of 128 participants were needed to detect a medium effect (d = .50) with power of .80. One-hundred and twenty-one individuals from the United States (66 Female, *M*_*Age*_ = 33.60, *SD*_*Age*_ = 12.71) participated in an online study via Mechanical Turk in exchange for .50 USD. Participants were 81.0% White, 3.3% Hispanic, 5.0% Black, 9.1% Asian, and 1.7% Other. All participants were again exposed a either an Inclusion or an Exclusion condition. As in Study 1a, participants’ sex did not interact with any of the results reported below and thus will not be discussed further.

#### Procedure

The procedure for this study was identical to Study 1 except the three people in the chat log were renamed as Sarah, Jessica, and Rebecca. Rebecca was either included or excluded during the conversation.

### Results and discussion

#### Manipulation check

We ran an independent sample t-tests on perceptions of inclusion for Rebecca (using our composite measure, α = .96). As we found in Study 1a, the manipulation was successful, *t*(119) = 12.28, *p* < .001, *d* = 2.28. In the inclusion condition, (*M* = 7.77, *SD* = 1.08) Rebecca was indeed perceived as being more included than when she was in the excluded condition (*M* = 3.41, *SD* = 2.48). Thus, our manipulation of social exclusion was again successful.

#### Internal/External attributions

We formed composite measures for the attributions made (α = .68). As in Study 1a, the same significant pattern emerged, *t*(119) = 4.46, *p* < .001, *d* = .82. When Rebecca was excluded (*M* = 6.58, *SD* = 1.73), perceivers made relatively more external attributions for her behavior than they did when she was included (*M* = 5.30, *SD* = 1.39). Thus, we replicated our findings from Study 1a.

#### Desire for affiliation

We also replicated our findings from Study 1a with respect to desire for affiliation, *t*(119) = 4.47, *p* < .001, *d* = .82. Again, when Rebecca was excluded (*M* = 5.89, *SD* = 1.87), perceivers had a greater desire to affiliate with her than when she was included (*M* = 4.49, *SD* = 1.52).

We combined the data from Study 1a and 1b and then conducted a series of 2 Inclusionary Status x 2 Target Sex x 2 Participant sex factorial ANOVAs on each of the three dependent variables (our manipulation check, external attributions, and desire to affiliate). In not one case did Target Sex or Participant Sex exert a main effect nor any interaction, at any level, with our dependent variables (all *Fs*<3.06, *ps>*.081.

#### Mediation analysis

**Fig 2:**
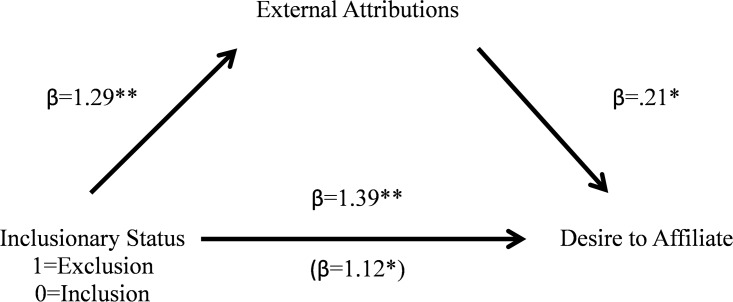
External attribution mediates the linkage between inclusionary status on desire to affiliate (Study 1b). **p< = .001, *p< = .05.

As in Study 1a, we again wanted to examine whether or not the extent to which individuals made more external attributions for Rebecca’s situation mediated the relationship between her exclusion and participants’ desire for affiliation. A bootstrapping mediation analysis [45] with 5000 iterations was conducted to examine this hypothesis. The experimental condition was coded as 1 (exclusion) or 0 (inclusion). The 95% confidence interval for the indirect path coefficient excluded zero (0.026 to 0.65), suggesting a significant indirect effect (see Fig 2 for betas and *p*-values).

Again, we found that our target’s exclusion (which was designed to be ambiguous as to its cause) was perceived as being due more to external factors than internal ones, and the extent to participants made these external attributions mediated their increased desire to affiliate with the excluded target. This replicates our findings from Study 1a, while also showing that the effect occurs for female targets as it did for male targets. In neither case did participant sex affect the results.

## Study 2

In Study 2, we wished to generalize our findings across another manipulation of exclusion. In this study, participants read about an employee at a company and some participants read that he was excluded by his peers (for no obvious reason). We hoped to demonstrate our findings are not context specific.

### Method

#### Participants and design

As in Studies 1a and 1b, a sample size of 128 participants was needed to detect a medium effect (d = .50) with power of .80. 137 individuals from the United States (77 female; *M*_Age_ = 32.35; *SD*_Age_ = 11.71) participated online to exchange for a payment of 0.20 USD. Participants were again recruited from Amazon’s Mechanical Turk. Participants were randomly assigned to either the ostracism or neutral control condition.

#### Procedure

After providing an online written consent, participants first exposed to an experimental manipulation of other’s ostracism or neutral experience by reading a brief description from one of our participants, Robert. In particular, all participants read that Robert was a new employee of a company. By random assignment, participants in the ostracism condition further read that Robert was ostracized by colleagues, and participants in the neutral control condition did not receive any information about Robert’s social relationship status. Afterwards, participants rated the extent to which they agreed with the three manipulation check questions: “Robert is included,” “Robert is excluded,” and “Robert is ignored, (1 = *strongly disagree*; 9 = *strongly agree*).” The scores were averaged to check the experimental manipulation (α = .93).

Next, participants completed the same three-item measure used previously that assessed participants’ attribution to Robert’s experience. Scores were reversed if necessary, and averaged; a higher score meant a higher external attribution (α = .83).

Finally, participants completed the same five-item measure used previously that assessed participants’ desire for affiliation with Robert. Scores were averaged; a higher score meant a higher proximity with Robert (α = .89). A debriefing followed.

### Results and discussion

#### Manipulation check

As expected, an independent sample t-test revealed that participants in the exclusion condition (*M* = 7.84, *SD* = 1.39) perceived Robert was ostracized more than participants in the neutral background condition (*M* = 4.39, *SD* = 1.33), *t*(135) = 14.83, *p* < .001, *d* = 2.54. Therefore, the manipulation was successful.

#### Internal/External attributions

As expected, another independent sample *t*-test revealed that participants in the exclusion condition (*M* = 5.91, *SD* = 2.01) made more external attributions for Robert’s experience than did participants in the neutral control condition (*M* = 5.10, *SD* = 1.59), *t*(135) = 2.61, *p* = .01, *d* = .45.

#### Desire for affiliation

Again, and replicating our prior studies, an independent sample t-test revealed that participants in the ostracism condition (*M* = 5.95, *SD* = 1.72) were more interested in interacting with Robert than participants in the neutral control condition (*M* = 4.85, *SD* = 1.65), *t*(135) = 3.82, *p* < .001, *d* = .65.

#### Mediation analysis

**Fig 3:**
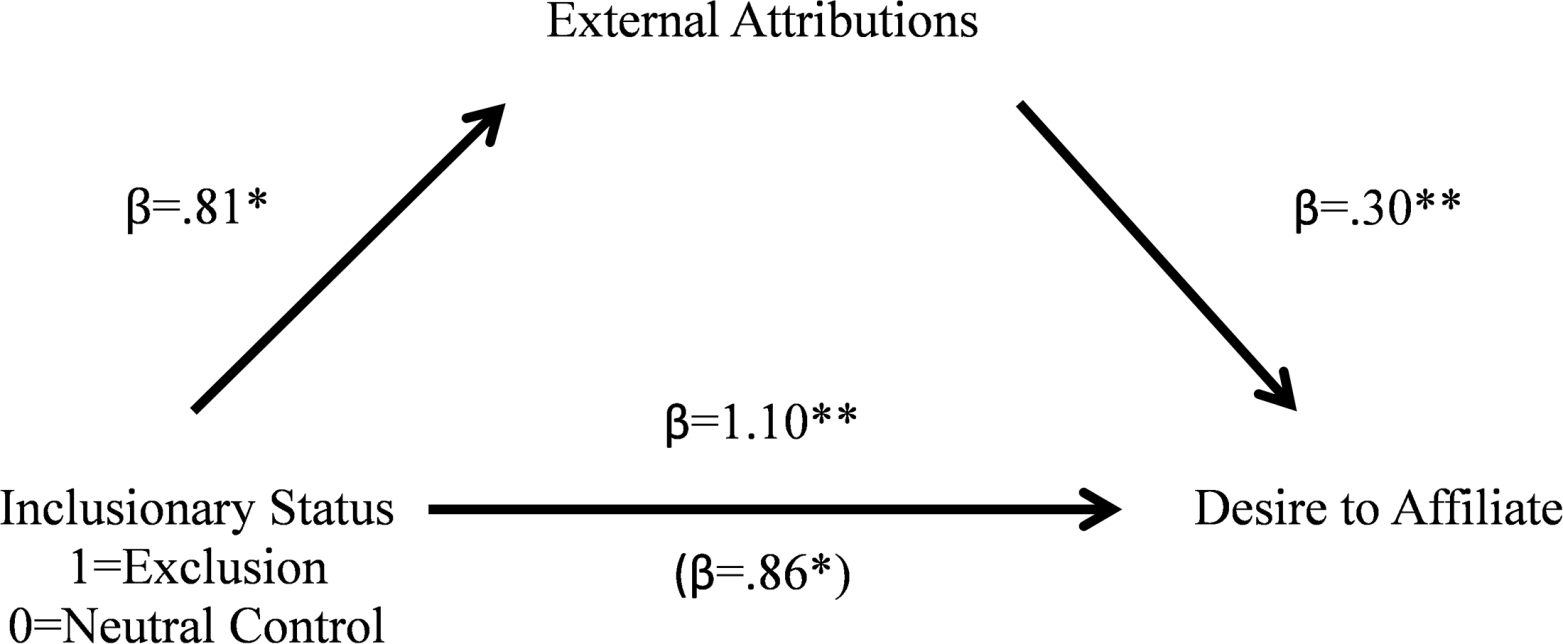
External attribution mediates the linkage between inclusionary status on desire to affiliate (Study 2). **p< = .001, *p< = .05.

A bootstrapping mediation analysis [45] with 5000 iterations was conducted to examine the hypothesis that a higher level of external attribution mediated the effect that reading about an ostracized target had on people’s desire for affiliation with the target. The experimental condition was coded as 1 (ostracism) or 0 (neutral background). The 95% confidence interval for the indirect path coefficient excluded zero (0.052 to 0.55), suggesting a significant indirect effect (see Fig 3 for betas and *p*-values). Thus, just as in our prior studies, the higher level of external attribution mediated the effect of ostracism background on people’s desire for affiliation.

We have now shown, in three studies using two manipulations of exclusion that in ambiguous situations where the cause of exclusion is not clear, individuals tend to make external attributions for exclusion and show an increased desire to affiliate with those excluded individuals. Further, in each of the three studies, we showed that the more individuals attributed exclusion to causes external to the target, the greater was their desire to affiliate with the target.

Some questions remain unanswered by our studies thus far. In three studies, we exposed participants to exclusion situations that happened with no apparent reason, so that participants develop their own attributions about the situation. As attributions people make to their observed exclusion have been found to be crucial in determining affiliative responses behaviors, it would be of theoretical and empirical interest to experimentally manipulate how people make attributions to their observed exclusion and further establish the causal relationship we imply.

Further, in our initial studies, we showed that external attributions mediated the relationship between perceived exclusion and desire to affiliate. However, while we believe external attributions are clearly a necessary component of this process, there must be some other psychological processes going on that results in a desire to affiliate; simply believing someone’s exclusion is the result of external factors should not alone account for a desire to affiliate with those individuals.

As stated previously, we believe that when someone makes an external attribution for someone’s plight of exclusion, this may lead the person to empathize with the target. In the first set of studies, we were able to show that exclusions with ambiguous antecedents were perceived as being externally caused which led to an increased desire to affiliate. However, these studies only showed that observers of causally unclear exclusion made more external attributions than observers of inclusion. To further these our hypothesis, it is necessary to directly examine whether observers of casually unclear exclusion show similar desire to affiliation with the target as observers of exclusion with clear external causes.

## Study 3

In Study 3, we included three conditions of exclusion. One of these was the original exclusion condition from Study 1a (now referred to as Exclusion Control). The other two conditions involved observing exclusion with clear external causes (i.e., Exclusion External condition) or internal causes (i.e., Exclusion Internal condition).

We then again asked questions to assess participants’ desire to affiliate with the target, predicting that participants should be more willing to affiliate with someone whose exclusion was due to external rather than internal factors. We further examined whether participants’ empathy for the target of exclusion mediated the aforementioned relationship between attributions and desire to affiliate.

Our primary focus was on the comparison between Exclusion Control condition and the Exclusion External condition. We expect that participants in both conditions would show similar levels of empathy concerns as well as desire for affiliation with the target of exclusion.

### Method

#### Participants and design

G*power analysis [41] showed that a sample size of 126 participants was needed to detect a medium effect (f = .30) with power of .85. One-hundred and forty-six (61 Female; *M*_*Age*_ = 33.95, *SD*_*Age*_ = 12.74) participated in an online study via Mechanical Turk. Participants were 79.5% White, 5.5% Hispanic, 4.1% Black, 7.5% Asian, and 3.5% Other. All participants were exposed to a 3-group design (Excluded Control, Excluded External, Excluded Internal).

#### Procedure

The procedure for Study 3 was very similar to Study 1a. All participants read a chat log. Participants were randomly assigned to one of three chat log manipulations. One of these was the original exclusion condition from Study 1a (now referred to as Exclusion Control).

In the Exclusion External condition, the chat log manipulation began identically to the other conditions but after Robert entered conversation in the chat room, an error message was shown appearing in the chat log. It read “ERROR MESSAGE: UNABLE TO DELIVER MESSAGE.” A second message appeared after Robert made another attempt: “ADMINISTRATOR MESSAGE: A TECHNICAL ISSUE HAS OCCURRED. ‘ROBERT’ WILL BE UNABLE TO SEND OR RECEIVE MESSAGES FOR THIS SESSION.” Thus, in this condition, Robert was excluded because of a computer error that prevented him from being included.

In the Exclusion Internal condition, the chat log manipulation again began the same but this time, Robert’s statements were rude and disparaging to others. For example, while the three men are tasked to discuss majors and career goals, Robert responds with “Yeah. I don’t care about these questions. You don’t care about my career goals and I sure as heck don’t care about yours. What is the point of this stupid assignment?!”

Following this, all participants completed the same manipulation check, measure of external attributions, and measures of desire for affiliation. Finally, all participants rated the extent to which they felt sympathetic, compassionate, warm, soft-hearted, and tender towards Robert (1-Not at all, 9-Very Much; α = .96; scale adapted from [13, 47]. All participants were then shown a debriefing and thanked for their time.

### Results and discussion

#### Manipulation check

To examine if our manipulation of attributions was successful, we performed a one way ANOVA on participants’ ratings of whether the causal agent of Robert’s plight was due to external factors (i.e., made external attributions). This revealed a significant effect, *F*(2,143) = 46.01, *p* < .001, η_p_^2^ = .39. Fischer’s LSD post hoc tests, indicated that all conditions were significantly different from each other (all *p*’s < .015); the Exclusion External condition was perceived as most due to external factors (*M* = 7.52, *SD* = 1.56), and then followed by the Exclusion Control condition (*M* = 6.51, *SD* = 1.73), and then the Exclusion Internal condition (*M* = 3.92, *SD* = 2.39). These results indicate that our manipulation of External and Internal attributions was successful.

#### Desire for affiliation

We next performed a one-way ANOVA on the measure of desire to affiliate with the target and found a significant effect, *F*(2,143) = 11.13, *p* < .001, η_p_^2^ = .14. Our results mostly supported our hypothesis; Fischer’s LSD post hoc tests revealed that Exclusion Internal (*M* = 3.57, *SD* = 2.25) was the least desirable as an affiliation partner as compared to both Exclusion Control (*M* = 5.51, *SD* = 2.13; *p* < .001) and Exclusion External (*M* = 4.78, *SD* = 1.74; *p* = .004). The latter two conditions, however, did not differ from each other (*p*>.08). While on its face, it may seem puzzling that these two groups did not differ, this may be due to the nature of our manipulation. While we clearly developed a manipulation in which the situation of Robert was perceived as being due to external factors (see previous analysis), we also confounded this with offering less opportunity for participants to learn information about Robert. Because of the computer error, Robert was unable to participate as much as even Robert did in the Exclusion Control condition, and thus the reason why there was no difference between this and the other exclusion condition may simply be due to participants having a dearth of information upon which to base a decision. Nonetheless, the decreased desire to affiliate in the Exclusion Internal condition as compared to both the of other Exclusion conditions (Control and External) is supportive of our hypothesis and still allows us to examine our remaining hypotheses.

#### Empathy

We again conducted a one-way ANOVA comparing the three condition on empathy for Robert and found a significant effect, *F*(2,143) = 22.05, *p* < .001, η_p_^2^ = .24. Fischer’s LSD post-hoc tests revealed that, consistent with our hypothesis, Exclusion Internal (*M* = 3.59, *SD* = 2.32) resulted in the least empathy as compared to both Exclusion Control (*M* = 5.59, *SD* = 2.01; *p* = .011) and Exclusion External (*M* = 6.28, *SD* = 1.95; *p* < .001). However, while Exclusion External had descriptively greater empathy than did Exclusion Control, it did not reach conventional levels of significance (*p* = .11).

#### Mediational analysis—Empathy

**Fig 4:**
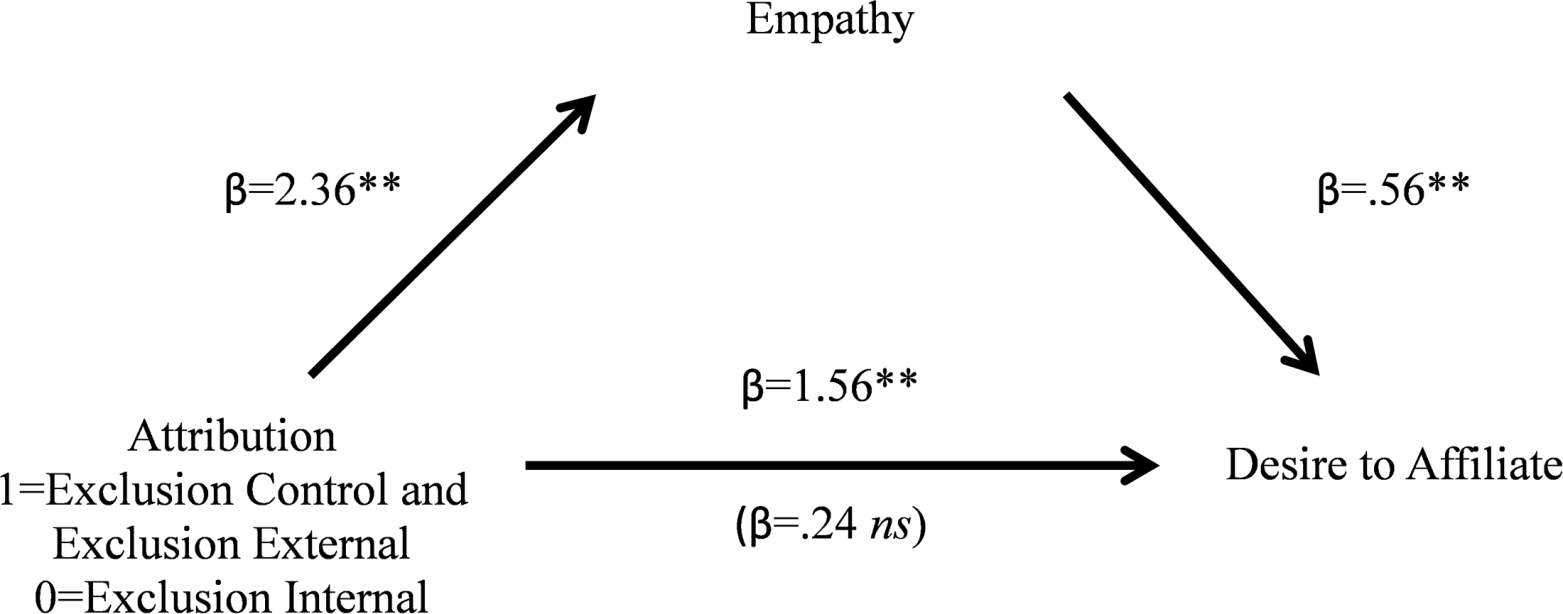
Empathy mediates the linkage between manipulated attribution on desire to affiliate (Study 3). **p< = .001, *p< = .05.

We wished to examine whether empathy mediated the differences in desired affiliation based on our manipulation of external attributions. Because the Exclusion Control and Exclusion External conditions did not differ on either our measure of desire to affiliate or empathy, we coded those two conditions as 1 and our Exclusion Internal condition as 0. The 95% confidence interval for the indirect path coefficient (5000 iterations) excluded zero (0.81 to 1.92), suggesting a significant indirect effect (see Fig 4 for betas and *p*-values). Empathy increased for the exclusion external attribution conditions relative to the internal attribution and this increase was associated with a greater desire to affiliate with Robert. When this was included in the model, the direct effect of manipulation attribution on desire to affiliate was no longer significant.

If we run the analyses keeping Exclusion Control and Exclusion External as separate predictors, we get the exact same results in the bootstrapping analysis. Rerunning the analysis using a Dummy Code of 1 (Exclusion Control) and 0 (Exclusion Internal) results in a 95% confidence interval (5000 iterations) that does not include 0 (.74–2.16). When using a Dummy Code of 1 (Exclusion External) and 0 (Exclusion Internal), we again find mediation through a bootstrapping analysis whereby the confidence interval does not include zero (.85–2.31).

In Studies 1a, 1b, and 2, we showed that when people read about a person who was excluded for ambiguous reasons, they tended to make more external attributions for their plight and this mediated their increased desire to affiliate with the target of the exclusion (relative to a target who was included). In this study, we directly manipulated the attributions people made for why the target was excluded. When the target was excluded for clearly situational reasons (i.e., a computer error that would not allow the target to participate), individuals showed similar desires to affiliate with the target as when we left the reasons for the exclusion ambiguous. However, when the target was a poor group member and acted inappropriately, people made internal attributions for his behavior and had far less desire to work with him. Importantly, these effects were fully mediated by the experience of empathy, our predicted mediator. We hypothesized that external attributions result in a greater experience of empathy for the target and this results in an increased desire to affiliate with the person. Indeed, when we manipulated attributions directly, we found evidence that supports this hypothesis.

Nonetheless, while these results are encouraging, this study alone does not fully support our hypothesis. Our contention is that perceiving an ambiguous exclusion should lead to greater external attributions that in turn should lead to an empathetic response, and this empathetic response should result in an increased desire to affiliate. To show this model in its entirety, we conducted Study 4.

## Study 4

In Study 4, we aimed to both replicate our prior findings that when perceiving ambiguous exclusions, individuals show an increased desire to affiliate with the target of the exclusion and that this is mediated by perceptions that the exclusion was situationally based (i.e., they make external attributions for the exclusion). Importantly, we also wished to show that it is not external attributions themselves which alone lead to a desire to affiliate; external attributions lead to an increased sense of empathy for the target which, in turn, results in a desire to affiliate.

### Method

#### Participants and design

A sample size of 146 participants was needed to detect a medium effect (d = .50) with power of .85. Two-hundred and thirty-five (126 Female; *M*_*Age*_ = 32.91, *SD*_*Age*_ = 11.77) participated in an online study via Mechanical Turk as in Study 1a. Participants were 82.1% White, 1.3% Hispanic, 7.7% Black, 6.4% Asian, and 2.5% Other. All participants were exposed to an Inclusion or Exclusion condition, just as in Study 1a.

#### Procedure

The procedure for Study 4 was identical to Study 1a with one addition. We included an empathy measure for the target. All participants rated their sympathetic concerns towards Robert as in Study 3.

### Results and discussion

#### Manipulation check

We conducted an independent sample t-test on the composite measure of inclusion (α = .96) to examine whether participants were sensitive to the differences in inclusionary status of Robert in the two conditions. This revealed a significant difference, *t*(233) = 16.64, *p* < .001, *d* = 2.16. When Robert was excluded (*M* = 3.22, *SD* = 2.42), he was perceived as being less included than he was in the inclusion condition (*M* = 7.68, *SD* = 1.63). Thus, our manipulation was successful.

#### Internal/External attributions

We next conducted an independent sample t-test on the composite measure of external attributions (α = .65). This too revealed a significant effect, *t*(233) = 6.48, *p* < .001, *d* = .84. When Robert was excluded (*M* = 6.57, *SD* = 1.73), perceivers made more external attributions than when he was included (*M* = 5.22, *SD* = 1.46). This replicates the effect found in the previous experiments.

#### Desire for affiliation

Again, an independent sample t-test revealed a significant difference between the two conditions in terms of our desire to affiliate composite measure (α = .83), *t*(223) = 3.08, *p* = .002, *d* = .40. Here, when Robert was excluded (*M* = 5.86, *SD* = 1.77), he was evaluated as a more desirable affiliation partner than when he was included (*M* = 5.18, *SD* = 1.59).

#### Empathy

An independent sample t-test on the composite empathy measure (α = .94) revealed the predicted difference between the two conditions, *t*(223) = 6.27, *p* < .001, *d* = 1.37. Here, when Robert was excluded (*M* = 6.54, *SD* = 1.94), he was empathized with to a greater extent than when he was included (*M* = 5.05, *SD* = 1.70).

#### Mediational analysis—External attributions

While our primary hypothesis in this study is about empathy and its relation to external attributions, we first wanted to see if we were able to replicate our mediational pattern from the previous studies in which external attributions mediate the relationship between inclusionary status and desire to affiliate. We again conducted a bootstrapping mediation analysis with 5000 iterations was conducted to examine this hypothesis. The experimental condition was again coded as 1 (ostracism) or 0 (inclusion). The 95% confidence interval for the indirect path coefficient excluded zero (.095 to 0.55), suggesting a significant indirect effect. As in previous studies, Inclusionary status was positively related to external attributions (β = 1.35, *p* < .001) which itself was positively related to desire to affiliate (β = .22, *p* = .002). While the direct effect of inclusionary status on desire to affiliate was significant (β = .68, *p* = .002), this effect becomes non-significant when including external attributions as a mediator (β = .39, *p*>.10). This is the same mediational pattern found in Studies 1a, 1b, and 2.

#### Mediation analysis—Empathy

We also wished to examine the mediating role of empathy. We conducted the same mediational analysis as above, but we included our empathy measure as our mediator. The experimental condition was again coded as 1 (ostracism) or 0 (inclusion). The 95% confidence interval for the indirect path coefficient excluded zero (.50 to 1.11), suggesting a significant indirect effect. As in previous studies, individuals who read about Robert being excluded were more interested in him as an affiliation partner (β = .68, *p* = .023). As expected, individuals who were excluded were shown greater empathy (β = 1.49, *p* < .001), and experiencing greater empathy led to a greater desire to affiliate with the target (β = .52, *p* < .001). When including the mediator, the effect of Inclusionary Status on desire to affiliate became non-significant (β = -.10, *p*>.61). This supports our predicted role of empathy.

#### Path analysis

**Fig 5:**
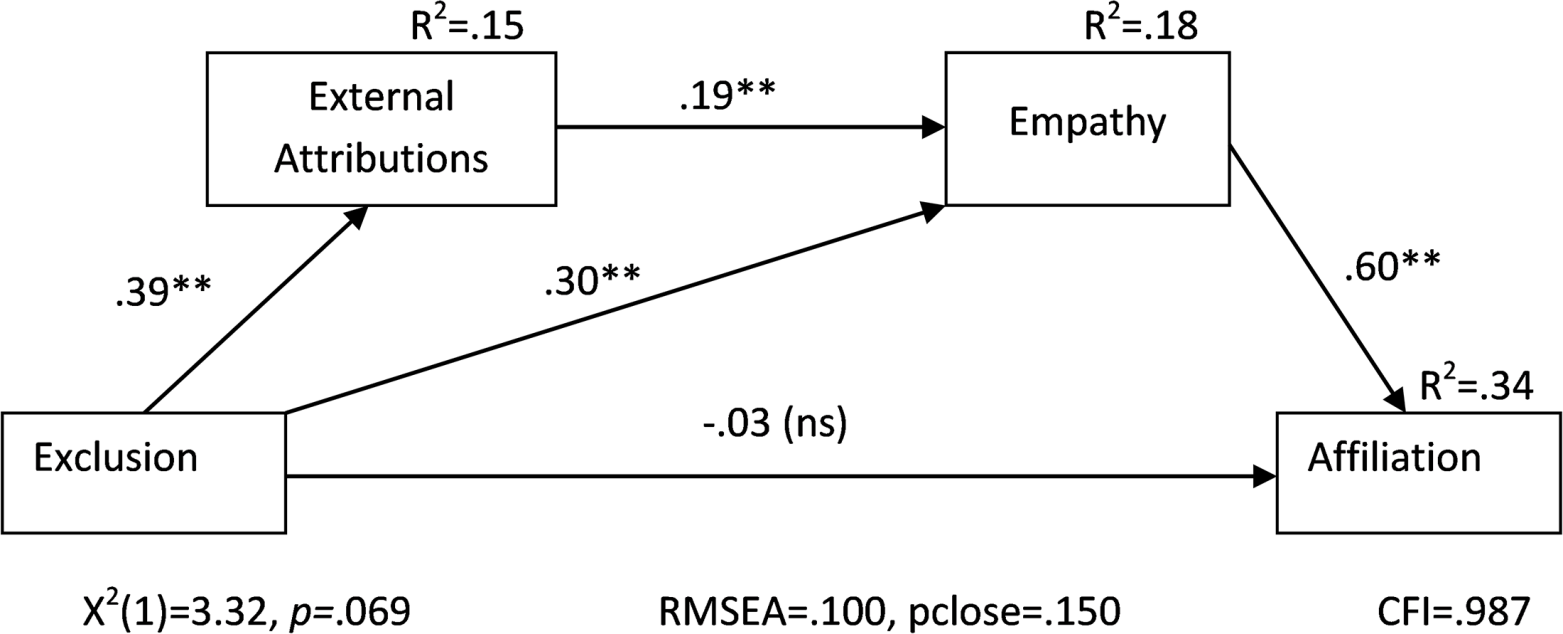
Path diagram of full empathy effect model (Study 4). **p< = .001, *p< = .05.

In the prior studies and the current analyses, results showed that either external attributions (Studies 1a, 1b and 2) or empathy (Studies 3 and 4) mediated the relationship between social exclusion and desire to affiliate with the excluded target. However, these analyses have not directly examined whether empathy accounts for the effect of attribution on desire for affiliation. To test this possibility, a path analysis model (Fig 5) consistent with our hypothesis was conducted.

In particular, the effect of external attributions was run through empathy (i.e., that external attributions would contribute to empathy which would independently mediate the effect of exclusion on affiliation). Model fit was good for this model on all three indices: Chi-square (χ^2^[1] = 3.32, *p* = .069), RMSEA (.10, 90% pclose = .150), and CFI (.987). Path estimates showed that the exclusion manipulation caused increases in both external attributions (β = .391) and empathy (β = .304). Additionally, external attributions related to higher amounts of empathy separate from that caused by the manipulation (β = .195). Finally, empathy was able to explain substantial amounts of variability in affiliation desire (R^2^ = .34) with higher empathy predicting more affiliation (β = .596). The direct path between exclusion and affiliation was non-significant (β = -.029, *p* = .616) showing that exclusion’s effect on affiliation occurs only indirectly through empathy and other processes that promote empathy such as making external attributions regarding the reason for the exclusion event (see Table 1 for direct and indirect effects).

**Table 1:** Standardized total, direct, and indirect effects for path model (Study 4).

			Direct Effect	Indirect Effect	Total Effect
Exclusion	→	External Attributions	.391	--	.391
Exclusion	→	Empathy	.304	.076	.380
Exclusion	→	Affiliation	-.029	.227	.198
External Attributions	→	Empathy	.195	--	.195
External Attribution	→	Affiliation	--	.116	.116
Empathy	→	Affiliation	.520	--	.596

Study 4, thus, offers further support for our hypothesis that when individuals perceive an ambiguous exclusion they make relatively external attributions, and this leads to an empathetic response which results in an increased desire to affiliate. Also, we provided support for our hypothesis that external attributions are a first step which then leads to an empathetic response.

## General discussion

Exclusion is a ubiquitous event in our lives, and much research has focused on how people respond when they are excluded. Here, we focused on how people perceive those who have been excluded. We hypothesized that when people perceive an ambiguous exclusion, they would make external attributions for the target’s plight and this would mediate their desire to affiliate with the victim of the exclusion. Further, we hypothesized that external attributions themselves would lead to an empathetic response which itself would mediate the initial exclusion—desire to affiliate relationship.

In five studies, we found support for our predictions. We first found that when people perceived an exclusionary experience for which there was no clear attribution to be drawn, they made more external rather than internal attributions and this resulted in an increased desire to affiliate with the excluded person (Studies 1a, 1b and 2). We found this occurred regardless of whether the target was male or female (Studies 1a and 1b). When we directly manipulated the attributions people would make for the exclusionary experience, either by making the exclusion due to a computer error (an external attribution) or due to the target being rude and non-cooperative (internal attributions), we again found that people were more willing to affiliate with the target when the exclusion was due to external rather than internal factors or unclear reasons (Study 3). Further, we showed that this effect was mediated by empathy; when the target was excluded due to factors outside of their person, perceivers felt empathetic for the target and this mediated their desire to affiliate (little empathy was experienced when the target’s exclusion was due to internal factors). Finally, using path analysis, we found support for our model: when perceiving an exclusion for which a cause is ambiguous, people make relatively external attributions and this then leads to an empathetic response which finally results in a greater desire to affiliate with the target (Study 4).

We believe this work is important for a number of reasons. First, relatively little work has been done on perceptions of those who are excluded, and this seems like a valuable and as of yet, somewhat unexplored area for future research. While much work has focused on the consequences of exclusion, perceptions of those who have been excluded, as well as the antecedents of exclusion have been left relatively unexamined. We believe the field would do well to study these other areas further as it seems there must be a host of moderators that should influence whether people want to approach or avoid those who have been excluded or give them a “second chance” as it were.

We also believe it is very interesting that the results of our studies seem to show a “default” response towards making more external relative to internal attributions when a person’s exclusion is causally unclear. As we stated in the introduction, there is good reason to think that people should be invested in understanding the causes of people’s exclusions. One could further have argued that, from an error management perspective [48], perceivers should have been especially cautious when observing the exclusion of another person, perhaps even defaulting to making internal attributions so as to avoid the potential danger of affiliating with a person who was indeed not a good group member. Our results clearly did not support that alternative hypothesis, and this is thus a very interesting and as of yet unanswered question—why did people make the less safe decision to make external attributions for the exclusion?

We can think of several potential reasons, all of which would make interesting avenues for future research. Of particular note, social inclusion is considered socially normative [49,50] and when this norm is unexpectedly violated, individuals find this particularly aversive [51]. While this pertains to those who are themselves excluded, when participants in one study were asked to ostracize another person as part of an experimental design, they reported feeling emotional distress when doing so [52]; when asked why they felt this way, many participants reported that it was upsetting to ostracize someone who did not deserve such treatment. Thus, it seems that individuals are not only sensitive to when norms of inclusion are violated with respect to the self, but also when it is violated for those who do not deserve such treatment. Thus, when participants observed our targets excluded without an obvious cause, the norm of inclusion may have led them to assume some external factor led to the precisely primarily because there was no apparent justification for violating the norm. It is also possible that perceivers made external attributions for the exclusion of our target because they were making internal attributions for the exclusionary behavior coming from those who engaged in the exclusion; it is possible that observing others exclude a person for no apparent reason led participants to “blame” the rejecters, and thus making an “external to the rejectee” attribution for the behavior. While this offers one other potential explanation. Future work should examine these issues.

In addition, Park and Park [53] found that observers dehumanize victims of social exclusion. In particular, after witnessing social exclusion via a ball-tossing game, participants judged the victim as less human, as indicated by traits capturing human nature and human uniqueness [54]. Interestingly, Park and Park [53] also found that participants (observers) judged the victim, relative to the perpetrators, as more agentic, more competent, warmer, more agreeable, and more moral. It is possible that witnessing an unjustified social exclusion threatens participants’ just world beliefs [55], leaving observers to engage in two distinct copying responses, including blaming the victim by denying their human qualities as well as empathizing and helping the victim. Future research can examine observers’ seemingly conflicting perceptions of the victims.

While we believe our work supports our hypothesis, there are of course limitations. One potential concern readers may have is whether or not the mediating factors in our model should be reversed; there is ample work showing that empathizing with others leads people to make more external attributions, focusing more on situational factors and less on dispositional ones [56–59]. In fact, empathizing with others has been shown to eliminate the actor-observer bias [60]. Thus, a reasonable question might be whether or not our model should be changed; rather than have perceptions of causally unclear exclusion leading to external attributions which results in an empathetic response that itself leads to a desire to affiliate, could it not be that perceiving an exclusion results in an empathetic response which in turn leads to external attributions resulting in an increased desire to affiliate. While we believe this model is potentially sound in terms of theory, the data from Study 4 does not support it; we conducted a path analysis in which we reversed the External Attributions and Empathy factors (and included a direct effect from Exclusion to External Attributions) to see if this model was better; in fact, it was a far worse model than our hypothesized one [Chi-square (χ^2^[1] = 82.36, *p* < .001), RMSEA (.590, 90%, p-close < .001), and CFI (.548)]. If we run the same path analysis without the direct effect of Exclusion on External Attributions, but with Empathy as the first step which then predicts External Attributions, we again get a model with very poor fit, [Chi-square (χ^2^[1] = 105.83, *p* < .001), RMSEA (.471, 90%, p-close < .001), and CFI (.423)]. We conducted a final model in which we again reversed Empathy and External Attributions (relative to our hypothesized model), but now, we included a direct effect of Empathy on Desire to Affiliate and removed the direct effect of Exclusion on Attributions, but this was also a poor model [Chi-square (χ^2^[1] = 23.46, *p* < .001), RMSEA (.310, 90%, pclose < .001), and CFI (.875)]. Given these alternative models, while it is reasonable to expect that empathy can lead to more external attributions for behavior, it does not appear to be able to explain our results in the present studies.

Another limitation is that we only examined affiliation intention and never actual affiliation or other associated behaviors. While behavioral intentions do tend to predict actual behavior (e.g., [61–64]), future work should examine whether perceivers who perceive others being unfairly excluded engage in behavioral responses of affiliation such as choosing them as a partner on a future task or allocating them greater resources in a resource sharing task. Other, more nonverbal measures of affiliation could also be examined such as seating distance and other immediacy behaviors (e.g., [65, 66]).

We also believe an important limitation of our work is that in all cases, participants essentially read about a situation in which someone was excluded. In Study 2, they read a vignette and were asked to imagine the situation (a commonly used manipulation). In Studies 1a and 1b, they read a chat room log of a supposed actual interaction. While this is closer to observing an actual exclusion taking place, insofar as they were ostensibly reading what was at some point a live interaction between people during which one person was excluded, generalizing from these studies to all social exclusion experiences should be done with caution. Future work should consider having people observe other exclusionary experiences (e.g., observing a Cyberball game [67]) and asked to complete the same set of measures we have here. In fact, Paolini and colleagues [68] have some evidence already in line with our predictions. In their work, participants observed a Cyberball game during with a target was ostracized or included by two “players” (the sources). Participants observing ostracism donated more money to the target and decreased donations to the sources, and this was mediated largely by negative impressions of the sources. This is suggestive of our hypothesis concerning attributions and empathy, though neither construct was assessed directly nor manipulated and no control condition was present to determine the direction of the effect. Nonetheless, it is a promising avenue for future work.

The consequences of social exclusion have been thoroughly researched in the past decade, but work on perceptions of those who have been excluded remains relatively unexamined. We believe this work demonstrates the importance of attributions in how we think, feel, and behave in response to observing the social exclusion of others. We believe future research in this area will open up new and potentially prosperous areas of work.
